# From Cullowhee Creek to Conley: genome sequence and annotation of a cluster DJ *Gordonia* phage

**DOI:** 10.1128/mra.01176-23

**Published:** 2023-12-22

**Authors:** Mindy N. Andro, AJ J. Cansler, Olivia S. Conley, Naidelyn O. Cruz, Joshua A. Dionne, Jordan I. Edwards, Daniel Furtuna, Carlee J. Green, Miriam Huber, Gunnar J. Hudson, Isabella R. Humphries, Amanda A. Karazin, Patrizia I. Mombille, Montana M. Pell, Gabriela Shickle, Zahria J. Shipp, Chloe A. Timmons, Jasmine I. Trembush, Cameron D. Turnmire, Naa Yemoley Yemofio, Mathew D. Burleson, Maria D. Gainey

**Affiliations:** 1 Department of Chemistry and Physics, Western Carolina University, Cullowhee, North Carolina, USA; DOE Joint Genome Institute, Berkeley, California, USA

**Keywords:** actinobacteriophage, cluster DJ

## Abstract

We report the discovery of *Gordonia* phage Conley, a siphovirus isolated from Cullowhee Creek in the Fall of 2022. The 60,078bp genome contains 90 predicted protein-coding genes all transcribed in the same direction and has been assigned to genetic cluster DJ based on gene content similarity.

## ANNOUNCEMENT

The Science Education Alliance Phage Hunters Advancing Genomics and Evolutionary Science (SEA-PHAGES) program has sequenced 4,561 actinobacteriophage genomes (1). This work has greatly expanded our understanding of the genetic diversity and evolution of actinobacteriophages (2). Our current report furthers this knowledge by reporting the genomic features of actinobacteriophage Conley.

**Fig 1:**
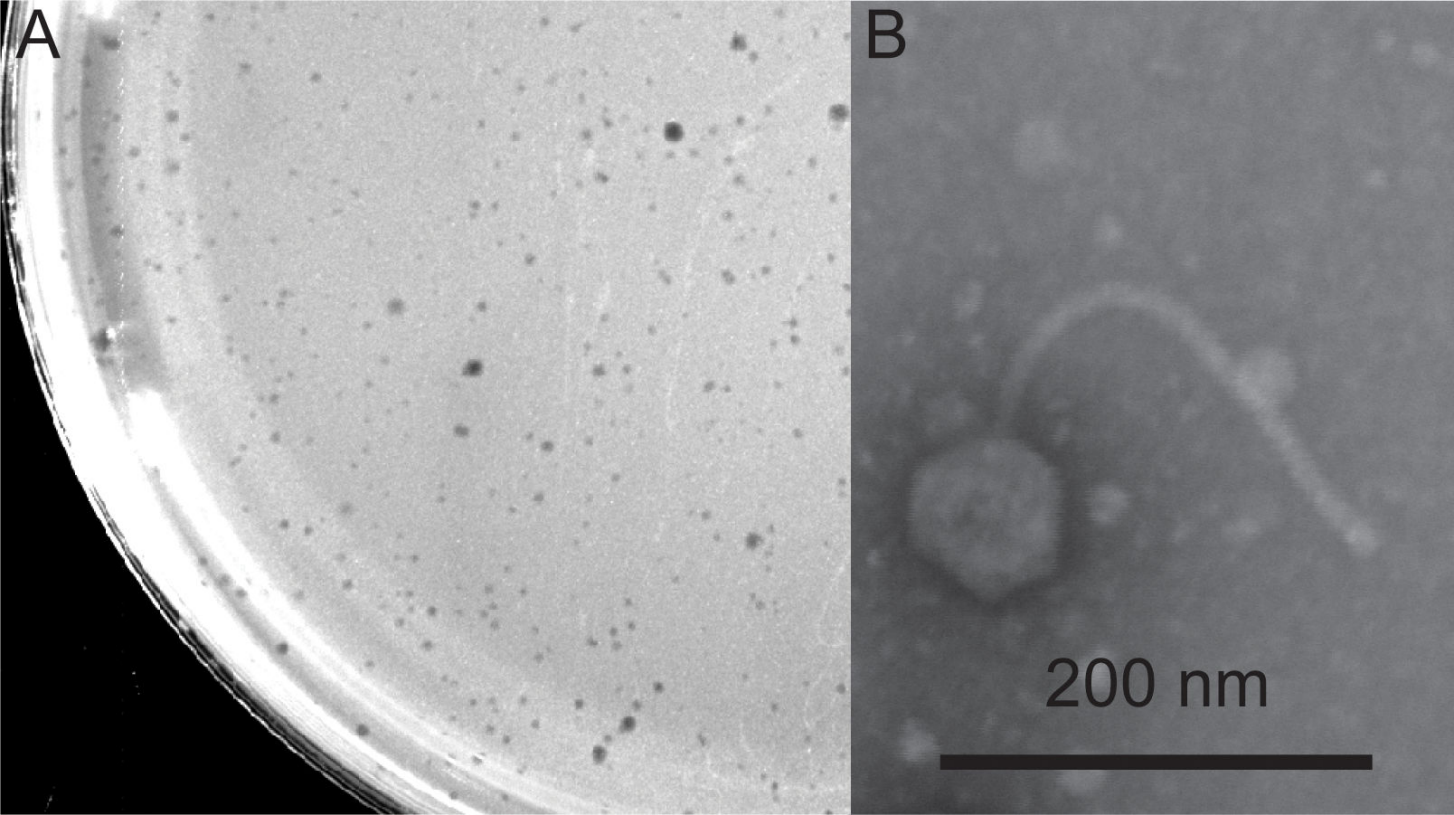
*Gordonia* phage Conley plaque and virion morphology. (**A**) Conley forms clear plaques on *Gordonia* top agar overlays when incubated for 48 h at 30°C. (**B**) Scanning transmission electron microscopy (STEM) of a Conley virus particle stained with 1% uranyl acetate. An Apreo 2 scanning electron microscope with a STEM 3 + detector in immersion lens mode with an accelerating voltage of 30 kV, a beam current of 0.20 nA, and a working distance of 7 mm was used to collect images of Conley virus particles (Scale bar = 200 nm).

Riverbed soil was collected from Cullowhee Creek (8/31/22), on the campus of Western Carolina University (35.310278 N, 83.18722 W). The sample was washed with peptone-yeast calcium (PYCa) media and filtered (0.22 μm) into fresh PYCa media containing *Gordonia rubripertincta* NRRL B-16540. After shaking incubation (48 h at 30°C), the sample was filtered (0.22 μm) and spotted onto PYCa plates containing *Gordonia rubripertincta* top agar overlays. After 48 h at 30°C, a clearing was observed indicative of phage infection. Phage Conley was purified through six rounds of plating, after which a lysate was prepared (https://seaphagesphagediscoveryguide.helpdocsonline.com/home). Conley plaques were clear (Fig. 1A). Scanning transmission electron microscopy (Fig. 1B) revealed a siphovirus morphology with 74.5 ± 1.5 nm isometric capsids and 264.0 ± 4.4 nm noncontractile tails (*n* = 4).

Genomic DNA was isolated from the lysate using a Promega Wizard DNA cleanup kit. DNA was prepared for sequencing using the NEB Ultra II library kit. In total, 546,261, 150b single-end reads were generated (1,297× genome coverage) using an Illumina MiSeq (v3 reagents). Raw reads were assembled into a single contig using Newbler v2.9 and checked for accuracy using Consed v.29 (3). Default parameters were used for all software in this manuscript unless otherwise specified.

The Conley genome is 60,078 bp long with 50.9% GC and has defined ends with a 9 bp 3′ overhang (CGCCGCCT). Based on gene content similarity (genes were grouped into phams using phamerator) of at least 35% to phages in the Actinobacteriophage database (1), Conley was assigned to cluster DJ, of which there are 36 members (4).

An automated annotation of the Conley genome was generated using DNA Master v2705 (http://cobamide2.bio.pitt.edu/) embedded with Genemark 2.5 p (5) and Glimmer 3.02 (6). Start sites were manually refined using Starterator v509 (http://phages.wustl.edu/starterator/), Phamerator v509 (7), and PECAAN v.20221109 (https://discover.kbrinsgd.org/evidence/summary). Putative functions were assigned using a combination of BLASTp (8) searches against the NCBI non-redundant and actinobacteriophage databases (1) and HHpred using PDB_mmCIF70, Pfam-A, UniProt-SwissProt-viral70, and NCBI_Conserved_Domains (CD) databases (9). No tRNAs were predicted using Aragorn v1.2.41 (10) and tRNAscanSE v.2.0 (*11*).

In all, 90 protein-coding genes were predicted, all transcribed in the same direction. In total, 22 were assigned a function. Structural genes are encoded on one half of the genome beginning at gene 13 (terminase large subunit), while DNA metabolism genes are located on the other half of the genome including a primase polymerase, a DNA helicase, and Cas4 family exonuclease (genes 61, 84, and 88, respectively).

Centrally located genes (34–55) frequently contained gaps (54–288 bp) preceding their start codons. A MEME suit 5.5.4 motif discovery search (12) revealed 5 instances of a 43 base motif containing a −10 sigma^70^ promoter sequence (TATAAT) in intergenic regions in this area as described for phage Vardy (13). Genes 26, 35, 68, and 79 are unique to Conley with no homologous genes found among actinobacteriophages.

## Data Availability

Conley is available in GenBank with Accession No. OR159671 and Sequence Read Archive (SRA) No. SRX21368094.
